# Three-Dimensional SnS Decorated Carbon Nano-Networks as Anode Materials for Lithium and Sodium Ion Batteries

**DOI:** 10.3390/nano8030135

**Published:** 2018-02-28

**Authors:** Yanli Zhou, Qi Wang, Xiaotao Zhu, Fuyi Jiang

**Affiliations:** School of Environmental and Materials Engineering, Yantai University, Yantai 264005, China; zhouyanli@ytu.edu.cn (Y.Z.); 18363898728@163.com (Q.W.); xiaotao.zhu@ytu.edu.cn (X.Z.)

**Keywords:** freeze drying, SnS, carbon nano-networks, lithium ion batteries, sodium ion batteries

## Abstract

The three-dimensional (3D) SnS decorated carbon nano-networks (SnS@C) were synthesized via a facile two-step method of freeze-drying combined with post-heat treatment. The lithium and sodium storage performances of above composites acting as anode materials were investigated. As anode materials for lithium ion batteries, a high reversible capacity of 780 mAh·g^−1^ for SnS@C composites can be obtained at 100 mA·g^−1^ after 100 cycles. Even cycled at a high current density of 2 A·g^−1^, the reversible capacity of this composite can be maintained at 610 mAh·g^−1^ after 1000 cycles. The initial charge capacity for sodium ion batteries can reach 333 mAh·g^−1^, and it retains a reversible capacity of 186 mAh·g^−1^ at 100 mA·g^−1^ after 100 cycles. The good lithium or sodium storage performances are likely attributed to the synergistic effects of the conductive carbon nano-networks and small SnS nanoparticles.

## 1. Introduction

Tremendous efforts have been made to search new anode materials to replace commercial graphite for lithium ion batteries (LIBs) to satisfy the ever-increasing requirement of high energy and power densities [1,2]. Metal sulfides as one of the promising anode materials in energy storage fields present so many advantages, such as the higher theoretical specific capacities compared with traditional commercial graphite (372 mAh·g^−1^), better conductivity, and smaller polarization in comparison with metal oxides [3,4,5,6]. However, the big volume changes during cycling and serious sulfur dissolution problems limit their potential applications. Currently, researchers have adopted two main strategies to enhance their electrochemical performances. One method is to synthesize nano-sized materials with special morphologies such as hollow nanospheres [7], flower-like structures [8,9], and so on. Another method is to introduce a carbon matrix to form composites [10,11,12]. For example, SnS nanorods/Carbon hybrid materials showed stable cycling performances in comparison with SnS nanorods for LIBs [10]. MWCNT@a-C@Co_9_S_8_ nanocomposites prepared by our group showed excellent electrochemical performances as anodes in LIBs, when cycled at 1 A·g^−1^, a high reversible capacity of 662 mAh·g^−1^ could be obtained after 120 cycles [12].

Except for LIBs, above strategies have also been used to fabricate various anode materials for sodium ion batteries (SIBs) [13,14,15]. For example, the composites of carbon-coated FeS on carbon cloth as anode materials delivered a reversible capacity of 365 mAh·g^−1^ after 100 cycles at 0.15 C [14]. Among these anode materials, SnS is a particularly interesting anode material for both LIBs and SIBs due to its high capacities (1022 mAh·g^−1^) and earth abundance [7,16]. Compared with SnS_2_, its lattice expansion is smaller, and it only contains two-structure phase reaction, corresponding to a combined electrochemical conversion and alloying mechanisms, which is different from those of transitional metal sulfides [10,16]. However, up to now, the composites of SnS decorated carbon nano-networks for both LIBs and SIBs have been few reported. 

Herein, a freeze-drying technique followed by an annealing process has been used to synthesize the three dimensional (3D) small SnS decorated carbon nano-networks. When evaluated as anode materials for both LIBs and SIBs, the composites demonstrate good cycling stability and rate performances. The facile synthesis method and good electrochemical performances indicate its potential applications in energy storage fields. 

## 2. Materials and Methods

### 2.1. Synthesis of SnS Decorated Carbon Nano-Networks

All of the chemicals were analytical-grade reagents and used without further purification. The typical preparation process for SnS decorated carbon nano-networks is as follows: First, SnCl_4_·5H_2_O (1.2 mmol), thiourea (2.4 mmol), C_6_H_8_O_7_·H_2_O (3.6 mmol) and NaCl (0.14 mol) were added into 50 mL deionized water, and then stirred for 30 min to form a clear solution. After that, the mixture was frozen for 24 h under vacuum using a freeze-drying machine. The obtained dry gel was ground to a fine powder and annealed at 720 °C for 2 h under Ar/H_2_ atmosphere. Finally, the composites were washed with deionized water to remove NaCl template to get the black powder, labeled as SnS@C. The single SnS was prepared under the same conditions with no added C_6_H_8_O_7_·H_2_O.

### 2.2. Materials Characterizations

The structures of all the samples were characterized by Bruke D8 Advance powder X-ray diffractometer (Karlsruhe, German) with Cu Kα (λ = 0.15418 nm)combined with the GSAS program for Rietveld refinement, and MicroRaman spectrometer using a laser of 473 nm as an excitation source (LabRAM HR Evolution, Kyoto, Japan). The related morphology and element composition (energy-dispersive spectra (EDS) and elemental mapping) of samples were obtained by high-resolution transmission electron microscope (HRTEM, JEOL-2100, Akishima, Tokyo, Japan) at an acceleration voltage of 200 kV and field-emission scanning electron microscope (FESEM, FEI Nova 450, Hillsboro, OR, USA). Thermogravimetric (TG) curves was carried out on a thermal gravimetric analyzer (NetzschSta 449F3, Selb, Germany) in O_2_ atmosphere from room temperature to 1200 °C with a heating rate of 10 °C·min^−1^. 

### 2.3. Electrochemical Measurements

The working electrode was prepared by mixing 70 wt % of active material, 20 wt % of acetylene black, and 10 wt % of carboxyl methyl cellulose (CMC) in deionized water, then above slurry was pasted on a clean copper foil, and dried in vacuum at 60 °C for 10 h. The final foil was roll-pressed and punched into discs with 12 mm. The mass loading of active material is about 1.0 mg·cm^−2^. The button cells were assembled in an argon-filled glovebox (H_2_O and O_2_ < 1 ppm), the lithium foil was used as the counter electrode, and a Celgard 2400 microporous polypropylene membrane was used as the separator. The electrolyte was composed of 1 M LiPF_6_ in ethylene carbonate (EC) and dimethyl carbonate (DMC) (1:1 *v*/*v*). For SIBs, the separator was glass fiber of Whatman GF/F and the electrolyte was 1 M NaClO_4_ in a mixture of EC-DEC (1:1 *v*/*v*) containing 2% fluoroethylene carbonate (FEC). Then, galvanostatic discharge and charge tests were carried out in the voltage range of 0.01–3 V on battery cyclers (Land CT2001A, Wuhan, China). Cyclic voltammetry (CV) curves were measured on an electrochemical workstation (CHI660E, Shanghai, China) over 0.01 to 3 V at a scanning rate of 0.1 mV·s^−1^. All the electrochemical tests were performed at 25 °C.

## 3. Results and Discussion

**Figure 1:**
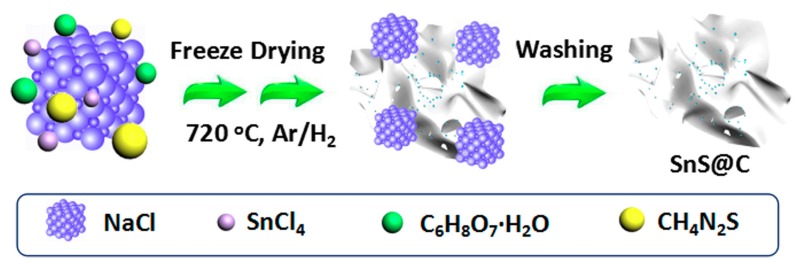
Schematic illustration of preparation process of SnS decorated carbon nano-networks (SnS@C) composites.

The preparation process of SnS@C composites are shown in Figure 1. First, SnCl_4_·5H_2_O, NH_2_CSNH_2_, and C_6_H_8_O_7_·H_2_O in the solution would chelate together to form complex, which could be easily adhered to the surfaces of formed NaCl crystals [17]. Second, the deionized water in the solution was gradually evaporated via a freeze-drying process to get a mixture. Finally, the mixture was annealed at 720 °C under Ar/H_2_ atmosphere for 2 h to obtain the SnS@C composites. During this heat treatment process, the carbon nano-networks were obtained from the high-temperature carbonization of citric acid, and nitrogen atoms in the carbon nano-networks was derived from thiourea. Meanwhile, Sn^4+^ could be partly reduced to Sn^2+^ by the formed carbon sources. Thus, S^2−^ coming from thiourea could react with Sn^2+^ to form SnS. More importantly, NaCl could be used as a framework template to control the morphology of final product, making for the formation of above 3D porous hierarchical composites.

**Figure 2:**
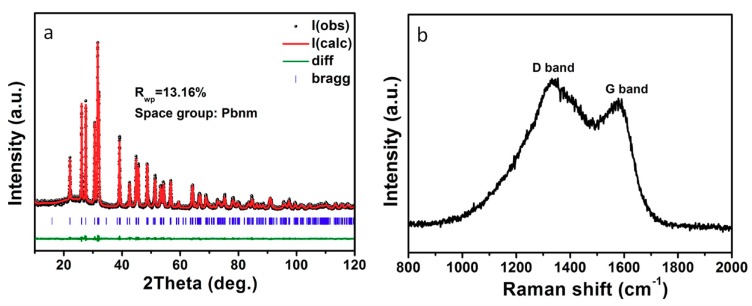
(**a**) X-ray diffraction patterns and Rietveld refinement results; and (**b**) Raman spectrum of SnS@C composites.

**Figure 3:**
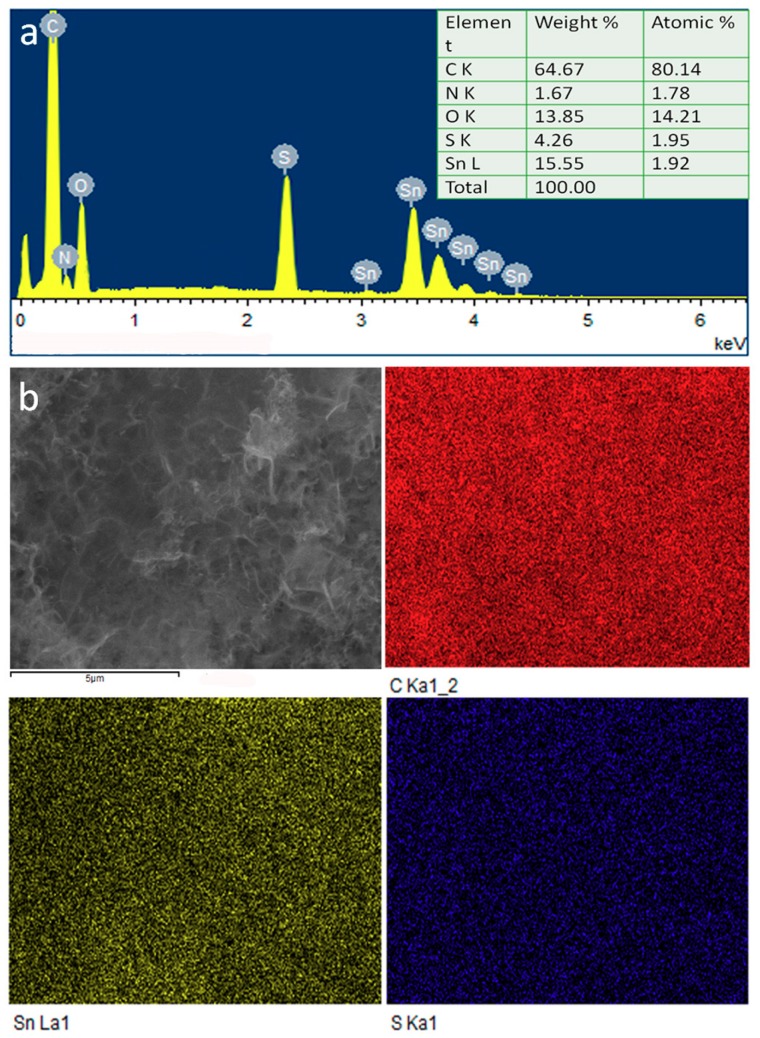
Energy-dispersive spectra EDS spectrum (**a**) and EDS elemental mapping (**b**) of C, Sn and S elements for SnS@C composites.

Figure 2a displays the X-ray diffraction (XRD) pattern and Rietveld refinement results of above SnS@C composites. It can be observed that all the sharp diffraction peaks could be indexed to orthorhombic SnS (JCPDS card No. 65-3766) [10]. No other impurity peaks are detected in the XRD patterns, indicating their high purity. The diffraction peaks of the N-doped carbon nano-networks are not been observed, implying its amorphous characteristic. To confirm the existence of carbon nano-networks, Raman spectrum of the composites was measured. As shown in Figure 2b, two distinct peaks at 1332 and 1567 cm^−1^ could be attributed to disorder-induced D band and graphitic G band of carbon material, respectively [18]. The value for D/G is estimated to ~1.2, suggesting that more disordered carbon exists in the carbon nano-networks. Moreover, the element analysis and element mapping for the composites are presented. As shown in Figure 3a, the SnS@C composites give a molar ratio of S/Sn of 1.95:1.92, close to the stoichiometric ratio of SnS. Besides S and Sn, carbon, nitrogen, and oxygen are also observed in the EDS spectrum, which might come from the carbon nano-networks. The existence of nitrogen element suggests that some N atom is successfully doped into the carbon nano-networks. Elemental mapping of C, S and Sn (Figure 3b) indicates that these elements are uniformly distributed in the SnS@C composites. Besides, the average carbon content in the SnS@C composites is estimated to 58.10% from the thermal analysis (TG) result (Figure S1).

**Figure 4:**
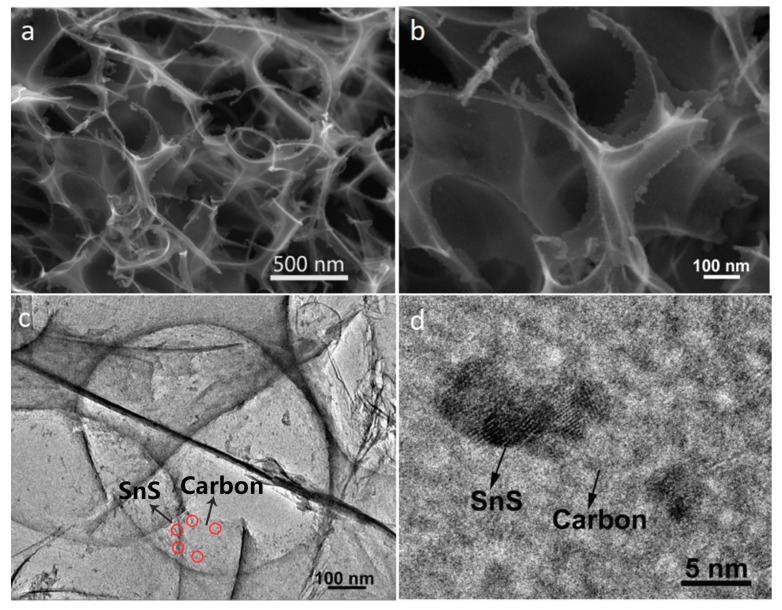
Scanning electron microscope (SEM) (**a**,**b**) and TEM images (**c**,**d**) of SnS@C composites.

Scanning electron microscope (SEM) and TEM images of SnS@C composites are shown in Figure 4. The SEM image in Figure 4a shows that a 3D porous network structure could be obtained, and the magnified SEM image demonstrates some nanocrystals are dispersed around the edge of the big pore in the carbon nano-networks (Figure 4b). TEM image in Figure 4c further clearly presents the magnified morphology, as can be seen, many small SnS nanocrystals with a particle size of below 5 nm adhere tightly to the surface of the carbon nano-networks, and the carbon nano-networks also have many large pores. Besides, a clear crystal lattice of SnS and amorphous carbon layer can be found from the HRTEM image (Figure 4d), indicating the composites of SnS and carbon nano-networks are formed. However, when the citric acid was not added to the mixture, the pure SnS nanoplates not nanocrystals were obtained (Figure S2).

**Figure 5:**
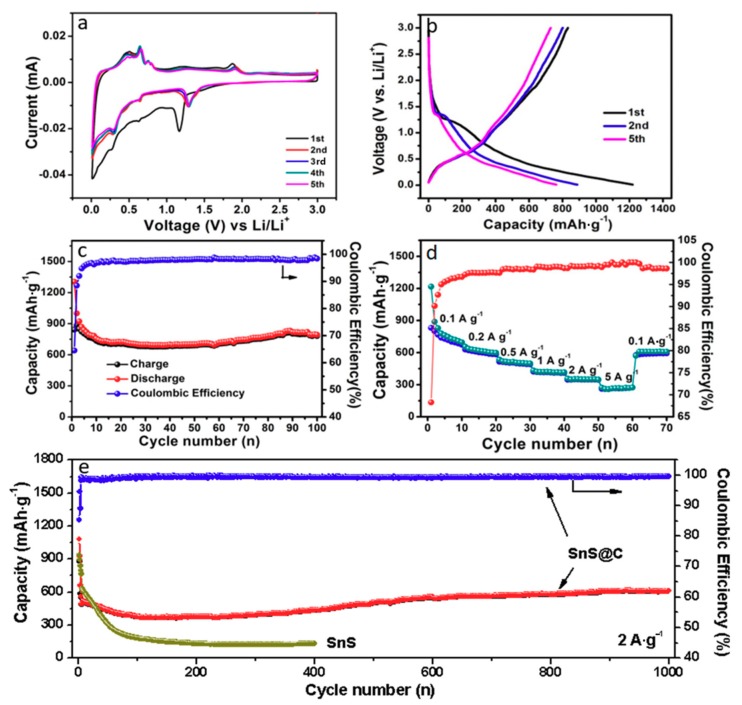
(**a**) CV curves; (**b**) discharge/charge profiles for the first, second and fifth cycle; (**c**) cycling performances at 100 mA·g^−1^ for 100 cycles; (**d**) rate capability at various current densities of SnS@C composites and (**e**) long-term cycling performances of SnS and SnS@C composites at 2 A·g^−1^ for LIBs.

The SnS@C composites were further used as anode materials to assemble half cells for potential application in energy storage fields. The related electrochemical performances of SnS@C composites for LIBs are shown in Figure 5. Figure 5a shows its CV curves in the voltage range 0.1–3 V with a scanning rate of 0.1 mV·s^−1^ at room temperature for the first five cycles, which present the similar electrochemical process with the literature [10,19]. The peak at about 1.2 V in the first cathodic scan corresponds to the reaction of SnS with Li^+^, SnS + *x*Li^+^ + *x*e^−^→Li*_x_*S + Sn. The wide small peak located at 0.63 V is ascribed to the alloying process of Li_x_Sn with the *x* range of 0.57–1.0 [20]. Another wide peak below 0.27 V can be assigned to the overlap of discrete lithium alloying process with the lithium content *x* range of 1.0–4.4 [20]. The two wide peaks below 1.2 V can correspond to the following reaction mechanism: Sn + *x*Li^+^ + *x*e^−^→Li*_x_*Sn. The peak near to 0 V is ascribed to the Li^+^ intercalation into carbon nano-networks. During the anodic scan, the peak at 0.2 V represents the Li^+^ deintercalation process from the carbon nano-networks [21]. Besides, there are four peaks corresponding to the de-alloying process of Li*_x_*Sn, indicating the discrete nature of the alloying and de-alloying process. The anodic peak at 1.88 V is attributed to the partial recombination of Sn^2+^ and S^2−^ to form SnS. The other wide peaks can correspond to the complicated de-alloying reaction. During the second cycle, the cathodic peak shifts from 1.2 to 1.28 V, and its peak position for the subsequent cycles is not changed. The anodic peak for the second cycle slightly shifts from 1.88 to 1.90 V, and the overlap of all the anodic peaks for the following cycles indicate good reversibility of SnS@C composites. Figure 5b shows the discharge/charge profiles of the composites for LIBs. For the first cycle, a sloping discharge plateau located at ~1.2 V can be observed, and the plateau for the subsequent cycles shifts to ~1.3 V due to the electrochemical activation of the material [19]. The charge plateau for all the cycles are overlapped, which is in good agreement with that of CV result. The discharge capacity for the first cycle is as high as 1218 mAh·g^−1^, however, the charge capacity is only 832 mAh·g^−1^, the irreversible capacity and low coulombic efficiency for SnS@C composites results from the initial irreversible lithium consumption and the inevitable formation of a solid electrolyte interface (SEI) layer [8]. The cycling performances of the composites are shown in Figure 5c. It can be seen that the reversible capacity of SnS@C composites can maintain at 780 mAh·g^−1^ after 100 cycles at a current density of 100 mA·g^−1^. The corresponding rate performances at various current densities are shown in Figure 5d. As can be observed, the average reversible capacities of SnS@C composites from 0.1 to 5 A·g^−1^ are 817, 603, 502, 414, 348, 265 mAh·g^−1^, when it returns to 0.1 A·g^−1^, the specific capacity could return to 594 mAh·g^−1^ after 70 cycles. Moreover, the long-term cycling performances of the composites at 2 A·g^−1^ are shown in Figure 5e. It can be seen that the specific capacity of SnS@C composites can still maintain at 610 mAh·g^−1^ after 1000 cycles, exhibiting excellent cycling stability, which is better than that of pure SnS and those of previous reports [22,23,24]. The excellent electrochemical performances are likely due to their specific structures. The 3D carbon networks can increase the conductivity of overall system, inhibit the volume changes of SnS upon cycling, and meanwhile suppress the dissolution of sulfur ion into the electrolyte. Moreover, the small particle size of SnS active materials can shorten the diffusion distance of Li^+^, and restrain the material pulverization. Therefore, the synergistic effect of SnS and 3D carbon networks contributes to the excellent electrochemical performances for LIBs.

**Figure 6:**
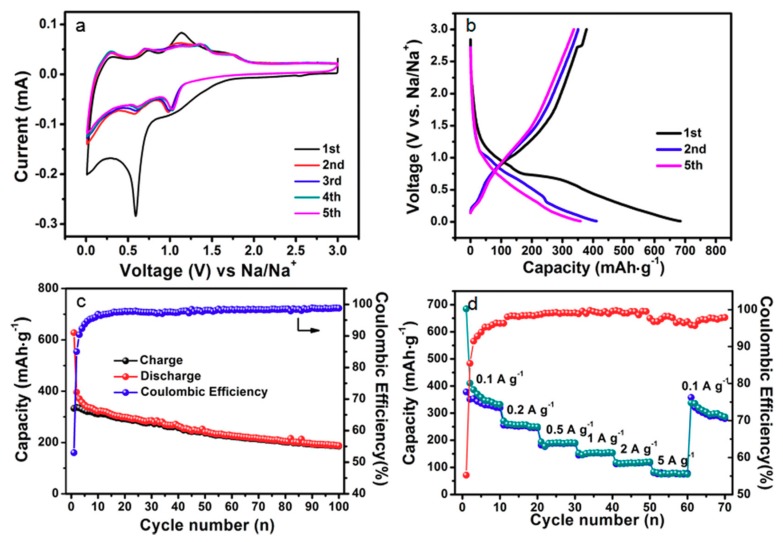
(**a**) CV curves; (**b**) discharge/charge profiles; (**c**) cycling performance at 0.1 A·g^−1^ for 100 cycles and (**d**) rate performances at different current densities of the SnS@C composites for SIBs.

Except for LIBs, the SnS@C composites were also used as anode materials for SIBs. The related electrochemical data are shown in Figure 6. Figure 6a shows its CV curves. In the first cathodic scan, the oxidation/reduction peaks located at 0.3–0.01 V are attributed to the deintercalation and intercalation process of Na^+^ into carbon nano-networks [25]. An obvious wide reduction peak located at 0.6 Vis attributed to the conversion of SnS to Sn and amorphous Na_2_S and the formation of a solid electrolyte interface (SEI) layer [26]. The oxidation peaks at 0.3 and 0.74 V are attributed to the formation of the reversible multi-step dealloying process of Na*_x_*Sn to Sn metal, respectively [26,27]. The two oxidation peaks observed at 1.14 and 1.72 V in the CV curves are attributed to the reversible conversion reaction from Sn to SnS [28]. The reduction peaks shift to 0.61 and 0.98 V for the following cycles, and five oxidation peaks located at 0.30, 0.73, 1.37, 1.72 and 1.11 V can be observed, which is similar with those of reports [26,27,28]. Except for the first cycle, the overlap of all of the cathodic and anodic peaks indicates good reversibility of SnS@Ccomposites.The discharge/charge profiles of SnS@C composites are shown in Figure 6b. It can be found that the voltage plateau for the first cycle is located at 0.93 V and 0.72 V, respectively, and the voltage plateau is not obvious for the following cycles. The first discharge and charge capacities of SnS@C composites are 685 and 378 mAh·g^−1^. The cycling performances of the composites are shown in Figure 6c. The reversible capacity can maintain at 186 mAh·g^−1^ at 100 mA·g^−1^ after 100 cycles. The capacity values for SIBs are obviously lower than those of LIBs, which is possibly due to the relatively large electrochemical equivalent of sodium and sluggish dynamics of SIBs. The related rate performance of the composites is shown in Figure 6d. As can be observed, the average reversible capacities from 0.1 A·g^−1^ to 5 A·g^−1^ are 339, 251, 187, 151, 116, 77 mAh·g^−1^, when the current density goes back to the original value of 100 mA·g^−1^, the average reversible capacity can go back to 302 mAh·g^−1^, manifesting good reversibility. Anyway, how to further increase the specific capacity and improve the cycling stability of SnS@C composites for SIBs would be a great challenge in the future.

## 4. Conclusions

In summary, the composites of small SnS nanoparticles attached to three-dimensional networks of carbon nano-networks have been fabricated by a freeze-drying and annealing process. As anode materials for both LIBs and SIBs, the composites exhibit good cycling performances and rate capacities. For LIBs, the reversible capacity of SnS@C composites can be maintained at 610 mAh·g^−1^ at 2 A·g^−1^ after 1000 cycles. For SIBs, it also exhibits stable cycling performances and the reversible capacity could be maintained at 186 mAh·g^−1^ after 100 cycles. The good lithium and sodium storage performances are attributed to the synergistic effect between carbon nano-networks and SnS nanoparticles. 

## Supplementary Materials

The following are available online at http://www.mdpi.com/2079-4991/8/3/135/s1, Figure S1: TG analysis of SnS@C composites, Figure S2: (a) XRD pattern and (b) TEM image of SnS nanoplates.
