# Supplementary material for: Three-Dimensional SnS Decorated Carbon Nano-Networks as Anode Materials for Lithium and Sodium Ion Batteries
Source: Nanomaterials (Basel). 2018 Feb 28;8(3):135. doi: 10.3390/nano8030135 (PMC5869626; doi:10.3390/nano8030135)
Supplement: Supplementary file 1 [file nanomaterials-08-00135-s001.pdf]

Supplementary materials

Three-Dimensional SnS Decorated Carbon Nano-Networks as Anode Materials for Lithium and Sodium Ion Batteries

Yanli Zhou, Qi Wang, Xiaotao Zhu and Fuyi Jiang *

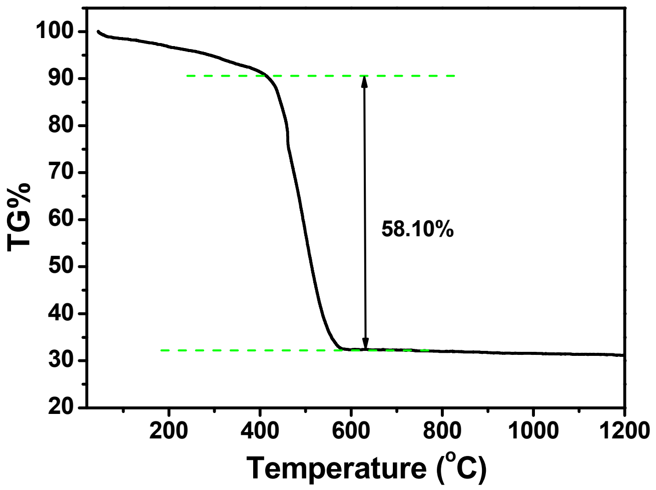

Figure S1. TG analysis of SnS@C composites.

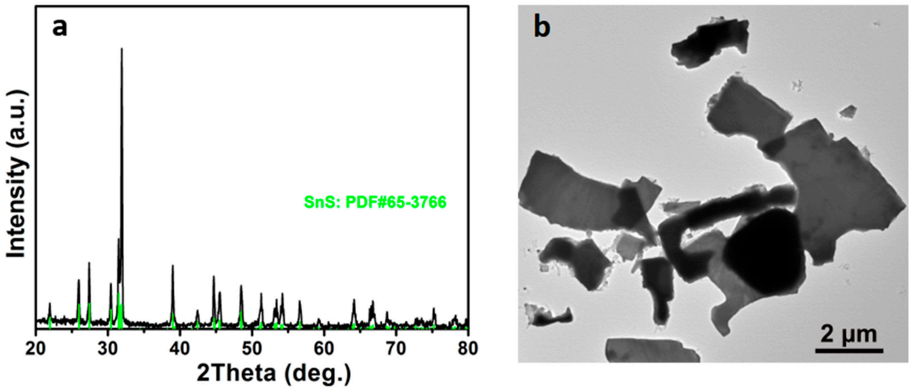

Figure S2. (**a**) XRD pattern and (**b**) TEM image of SnS nanoplates.
